# Efficacy of dapoxetine treatment in Chinese patients with premature ejaculation and possible factors affecting efficacy in the real-world practice

**DOI:** 10.1186/s12894-020-0580-3

**Published:** 2020-02-03

**Authors:** Jing Peng, Dong Fang, Huixi Li, Yuan Tang, Yiming Yuan, Wanshou Cui, Bing Gao, Hongjun Li, Zhichao Zhang

**Affiliations:** 1 Andrology Center, Department of Urology, Peking University First Hospital, Institute of Urology, Peking University, No. 59A, Di’anmen West St., Xicheng District, Beijing, 100034 China; 2 Department of Urology, Peking Union Medical College Hospital, Peking Union Medical College, Chinese Academy of Medical Sciences, No. 1 Shuaifuyuan, Dongcheng District, Beijing, 100730 China

**Keywords:** Premature ejaculation, Dapoxetine, Sexual dysfunction, Efficacy

## Abstract

**Background:**

The treatment effect of dapoxetine in real-world practice is not well established. This study was to investigate the factors influencing efficacy of dapoxetine for the treatment of Premature ejaculation (PE) in the real-world setting.

**Methods:**

Altogether 154 patients were followed up between Jan 2015 and Dec 2015. The clinical global impression of change (CGIC), premature ejaculation profile (PEP), the estimated intravaginal ejaculation latency time (eIELT) and estimated number of intravaginal thrusts before ejaculation (NITBE) were collected. The clinical characteristics of patients with CGIC = 0 and CGIC≥1 were compared.

**Results:**

After 4 weeks treatment, an obvious improvement compared with the baseline was found regarding mean eIELT (2.4 ± 1.6 min vs 1.0 ± 0.7 min, *P* < 0.001) and mean NITBE (85.9 ± 61.9 times vs 37.4 ± 28.6 times, *P* < 0.001). The proportion of patients with a self-evaluation of at least “slightly better” and were categorized into “CGIC≥1” group was 70.1%. There were significant differences between patients in the “CGIC = 0” and “CGIC≥1” groups regarding mean NITBE (*P* = 0.010) and PEDT (*P* = 0.009) score at baseline. The adverse effects were acceptable.

**Conclusion:**

Dapoxetine was well-tolerated and improved the sexual satisfaction of patients with PE. The severity of PE based on PEDT and NITBE suggest that there could be an effectiveness change with dapoxetine use in real-world practice.

## Background

Premature ejaculation (PE) is one of the most of prevalent male sexual disorders, with a reported incidence of 21–33% in some populations [1, 2]. Dapoxetine hydrochloride, a short-acting selective serotonin reuptake inhibitors (SSRIs), is the currently the only on-label oral treatment [3]. This on-demand agent has been proved to increase the quality of life for the patient and their sexual partner by a pooled analysis of five randomized, placebo-controlled, phase 3 clinical trials (*N* = 6081) [4] and by a critical review [5].

Most data on the efficacy of dapoxetine are derived from clinical trials involving Western participants, and results from clinical trials may not be consistent with the results observed in the real-world practice. Clinical trials follow strict protocols involving patients with pre-defined inclusion criteria and results are obtained under ideal conditions. In contrast, clinical practice has several associated challenges including low rates of compliance, economic considerations, and a lack of awareness of the condition by the spouse, and these factors may influence the real-world utility of treatments for PE. In real-world setting dapoxetine treatment discontinuation was high. Mondaini et al. [6] reported that 68.7% of patients would discontinue dapoxetine treatment in a short time. The main reason was effect below expectations. It is important to achieve a reasonable effect in a short term.

Furthermore, stopwatch-determined intravaginal ejaculation latency time (IELT) is the most common measure to diagnose and evaluate treatment efficacy in clinical trials, which is not appropriate in real world practice. In the clinical setting, stopwatch-determined IELT is substituted by self-estimated IELT, which is often overestimated by patients [7].

In this study, we sought to evaluate the treatment effect of dapoxetine on Chinese patients with PE in the real-world practice, and to investigate factors influencing treatment efficacy of dapoxetine.

## Methods

### Patients inclusion

This open-label, retrospective and observational study included patients with PE who accepted dapoxetine treatment in real-world practice. All patients reported short intravaginal latency, and at least ‘moderate’ distress or interpersonal difficulty relating to their PE at baseline. To be included in the study, patients had to be over 18 years, and were required to be in a heterosexual, stable, and monogamous sexual relationship with the same partner for at least 6 months. The Premature Ejaculation Diagnostic Tool (PEDT) was used to diagnose PE [8] and subjects with a PEDT score ≥ 11 were included. The linguistic validation of PEDT questionnaire has been performed and the Chinese version was used [9, 10]. Patients were excluded from the study if they experienced primary erectile dysfunctions, engaged in sexual intercourse less than once per week, abused alcohol or illicit substances, had a history of medical or psychiatric illness, or if their partner experienced any sexual dysfunction. The study received the ethics approval by committee of Peking University First Hospital. All patients agreed and signed the informed consent, that their information (including clinical information and surveillance) would be collected for scientific study and by published in professional medical journals.

### Patients treatment and outcome measures

After a 4-week run-in period, patients were asked to take dapoxetine 30 mg 1–3 h before planned sexual intercourse. No other PE therapies were provided during the study period. Patients were assessed after 4 weeks of treatment, and were required to engage in sexual intercourse at least 6 times during the 4-week study period.

All measures including eIELT and estimated number of intravaginal thrusts before ejaculation (NITBE) were evaluated at baseline and at week 4. The definition of NITBE was the frequency of penile moving forward and backward in female vaginal. The patients were demanded using the similar frequency and depth of insertion as before and recorded the average NITBE.

Patient were required to answer the question from CGIC “Compared to the start of the study, would you describe your premature ejaculation problem as much worse, worse, slightly worse, no change, slightly better, better, or much better?” and the answers were scored from 0 to 3 (0: no change, 1: Slightly better, 2: better, 3: much better) respectively. Patients were also assessed based on the Premature Ejaculation Profile (PEP) [11]; a validated tool that includes measures of perceived control over ejaculation, satisfaction with sexual intercourse, ejaculation-related personal distress, and ejaculation-related interpersonal difficulty. Part of the database is attached in Additional file 1.

Patient Health Questionnaire (PHQ-9) and General Anxiety Disorder-7 (GAD-7) questionnaire were used to evaluate patients’ depression and anxiety [12, 13]. The linguistic validation of PHQ-9 and GAD-7 questionnaires have also been performed and the Chinese versions were used [14, 15].

### Statistical analysis

Statistical analysis was carried by using SPSS 20.0 (IBM Corp, Armonk, NY, USA). Continuous variables normally distributed were expressed as mean ± SD, otherwise median and range (min, max) were used. For categorical variables, frequency and percentile were used to describe the data. Patients were categorized into two groups based on their answer to CGIC after 4 weeks treatment: “CGIC = 0” group vs “CGIC≥1”.

Clinical characteristics between the two groups were compared using Chi-square or Mann-Whitney test. The comparison of IELT and NITBE before and after treatment was performed using paired T test. All statistical tests were two-tailed and a *P* value of less than 0.05 was considered as statistically significant. The receiver-operating curve for treatment effect was also analyzed.

## Results

**Fig. 1:**
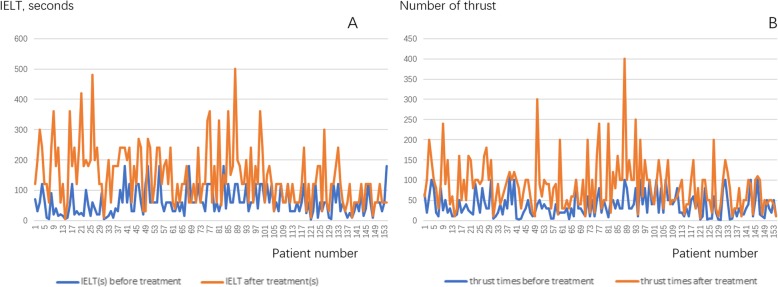
IELT distribution (**a**) and thrust times distribution (**b**). IELT: intravaginal ejaculation latency time

One hundred seventy-two patients with PE received dapoxetine therapy (30 mg on-demand) for 4 weeks were included in this study. One hundred fifty-four patients were followed up and their data were analyzed and compared at baseline and after dapoxetine treatment. The mean age was 32.5 ± 6.8 (range: 21–61) years. After 4 weeks treatment, there was an obvious improvement compared with the baseline regarding mean eIELT (2.4 ± 1.6 min vs 1.0 ± 0.7 min, *P* < 0.001) and mean NITBE (85.9 ± 61.9 times vs 37.4 ± 28.6 times, P < 0.001) (Fig. 1).

**Fig. 2:**
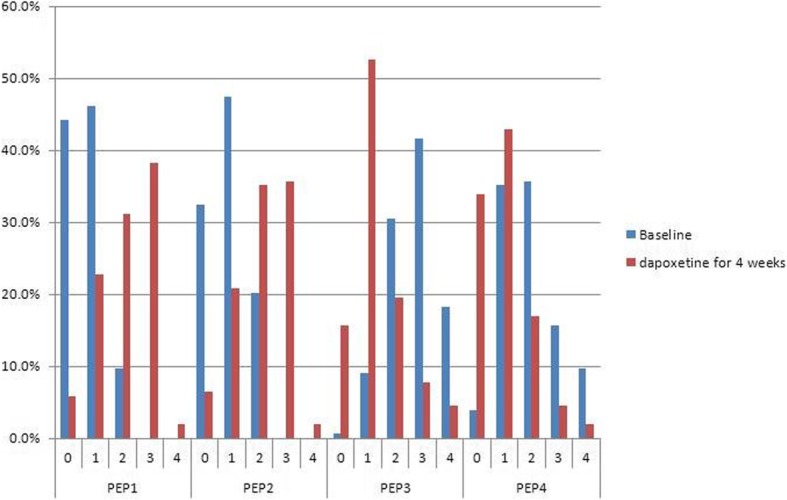
Comparison of PEP before and after dapoxetine treatment. All PEP measures showed significant improvements with dapoxetine treatment at week 4 vs. baseline. PEP: premature ejaculation profile

Substantial improvements were found in all PEP measures (Fig. 2). At baseline, 9.7 and 0% of patients reported fair or good control over ejaculation which increased to 31.2 and 38.3% at week 4, respectively (*P* < 0.001). At baseline, 20.1 and 0% of patients reported fair or good satisfaction with sexual intercourse which increased to 35.1 and 35.7% at week 4, respectively (*P* < 0.001). At baseline, 30.5 and 41.6% of patients reported moderate or quite a bit of ejaculation-related personal distress which decreased to 19.5 and 7.8% at week 4, respectively (*P* < 0.001). At baseline, 35.7 and 15.6% of patients reported moderate or quite a bit of interpersonal difficulty which decreased to 16.9 and 4.6% at week 4, respectively (*P* < 0.001).

**Fig. 3:**
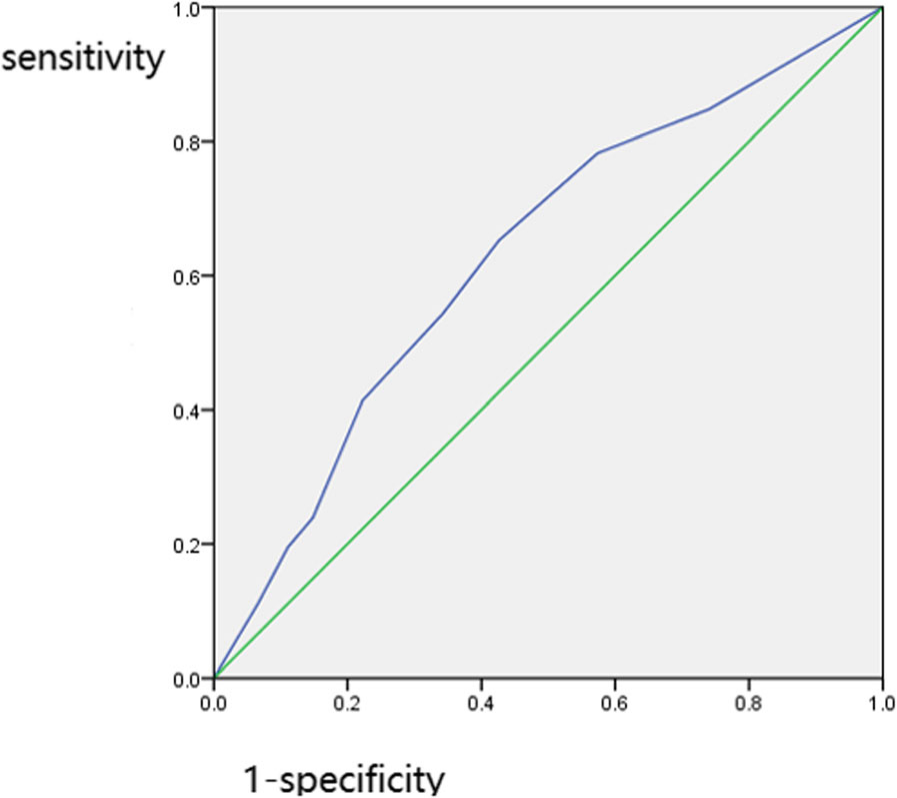
The receiver-operating curve (ROC) of PEDT for treatment effect. Note: since higher PEDT score was related to poor treatment effect, the ROC curves are showing the prediction of CGIC = 0, NOT CGIC≥1 (better results). PEDT: Premature Ejaculation Diagnostic Tool; CGIC: clinical global impression of change

Overall, 108 patients (70.1%) answered the CGIC question with “slightly better”, “better” or “much better”, and were categorized into “CGIC≥1” group (Table 1). Regarding the clinical characteristics between the two groups, the mean NITBE at baseline was 28.0 ± 22.0 and 41.5 ± 30.1 times (*P* = 0.010) and mean PE severity at baseline measured by the PEDT was 15.7 ± 2.7 and 14.5 ± 2.6, respectively (*P* = 0.009), which indicated that the severity of PE and reduced NITBE prior to treatment may be associated with reduced treatment effect. A ROC curve was made to illustrate the relationship between PEDT scores and treatment effect (Fig. 3). Based on Youden index, the best cut-off value was 14.5, with sensitivity 65.2% and specificity 57.4%. A PEDT score of lower than 14.5 points would indicate a possibly better treatment effect. Notably, no significant difference in terms of baseline eIELT was found between the two groups (56.3 ± 44.4 vs 63.0 ± 42.5, *p* = 0.262). Baseline characteristics were also similar, including age, PE duration, previous treatment, and intercourse frequency (all *p* > 0.05).

**Table 1:** Possible factors affecting the efficacy of dapoxetine 30 mg treatment for 4 weeks

Parameters	CGIC-C		*P*
	0 (*n* = 46)	≥1 (*n* = 108)	
Age (yeas, mean ± SD)	33.8 ± 8.1	31.9 ± 6.1	0.213
Marital Status: Married	88.6%	84.5%	0.508
PE duration (years, mean ± SD)	6.4 ± 5.7	8.2 ± 6.8	0.058
Type of PE (% of primary PE)	84.8%	70.4%	0.060
Percentage of Intercourse frequency ≥ 2 times	43.4%	53.7%	0.245
Percentage of CP	41.3%	41.7%	0.967
Percentage of previous SSRI use	10.9%	18.5%	0.239
Percentage of previous Effective SSRI use	10.0%	28.6%	0.097
Percentage of history of Circumcision or penile dorsal nerve transection	28.3%	34.3%	0.467
PHQ-9(median [min, max])	6.0 [0, 27]	6.0 [0, 26]	0.835
GAD-7(median [min, max])	4.0 [0, 25]	3.5 [0, 21]	0.253
IELT (Seconds, mean ± SD)	56.3 ± 44.4	63.0 ± 42.5	0.262
NITBE (times, mean ± SD)	28.0 ± 22.0	41.5 ± 30.1	0.010^*^
PEDT (mean ± SD)	15.7 ± 2.7	14.5 ± 2.6	0.009^*^

*CGIC* Clinical Global Impression of Change, *CP* chronic prostatitis, *ED* erectile dysfunction, *GAD-7* General Anxiety Disorder Questionnaire, *IELT* intravaginal ejaculatory latency time, *NITBE* number of intravaginal thrusts before ejaculation, *PE* premature ejaculation, *PEDT* Premature Ejaculation Diagnostic Tool, *PHQ-9* Patient Health Questionnaire-9 items, *SD* standard deviation, *SSRI* selective serotonin reuptake inhibitors

^*^statistical significance

Regarding adverse effects, 21 patients (13.6%) eventually discontinued dapoxetine treatment, and the reasons included lack of efficacy (*n* = 13), side effects (*n* = 5), and low frequency of sexual intercourse (*n* = 3). Of the patients who completed the 4-week treatment regimen, 72.2% would like to continue dapoxetine therapy. Treatment emergent adverse events (TEAEs) were reported in 32 patients (20.8%), that mainly included headache (7.1%), dizziness (7.1%), nausea (4.5%), somnolence (1.9%) and nasal congestion (1.9%). Most TEAEs were mild or moderate in severity and resolved without treatment.

## Discussion

PE can interfere with sexual satisfaction, leading to decreased quality of life for the patients and their partners [16–18]. The mechanism for developing PE has not been fully elucidated, especially considering the role of physiological components. SSRIs had been introduced to the treatment of PE following the psychopharmacological studies regarding pathways that control ejaculation [19, 20]. SSRIs could inhibit neuronal re-uptake of serotonin and subsequent potentiation of serotonin activity, and since the serotonergic neurotransmission is related to the pathways which control ejaculation, the delayed ejaculation is a commonly reported side effect [21, 22]. However, these compounds are off-label, long-acting and not ideal for on-demand use.

Based on a pooled analysis of five randomized, placebo-controlled, phase 3 clinical trials, dapoxetine, given on-demand, could delay ejaculation approximately 2.5–3 fold and the therapy efficacy were better in those with lower baseline IELT. After 12 weeks 30 mg or 60 mg dapoxetine treatment, 62.1 and 71.7% of subjects reported that their PE was at least “slightly better”, while in the control group this proportion was only 36.0% (*P* < 0.001 for both). Dapoxetine also improved overall sexual satisfaction, and reduced mental consequences including personal distress and interpersonal difficulty [4].

However, clinical data on dapoxetine treatment in real world practice is limited. Jiann et al. [23] reported the satisfaction rate and response rate were 45.0 and 74.6%, respectively. We found dapoxetine treatment was well-accepted by patients with PE and 70.1% of patients reportedly responded to dapoxetine treatment on demand.

In this study, a total of 70.1% of patients reported that their PE was at least “slightly better” with dapoxetine treatment, and 40% of patients evaluated their treatment effect as “better”. Yang et al. [24] reported similar results in Chinese men with PE. The rate of improvement and excellence with dapoxetine 30 mg was 63.5 and 36.5%, respectively. This reflects what was reported in five combined phase 3 trials of 6081 patients, whereby 62.1% of subjects taking dapoxetine 30 mg reported that their PE was at least “slightly better” and 30.7% reported “better” compared with 13.9% in the placebo arm (*P* < 0.001) [4]. Only those who reported that their PE was “better” were satisfied with sexual intercourse.

Dapoxetine was well-tolerated with 72.2% of patients stating that they would continue dapoxetine therapy. In our study, 64.4% of patients who discontinued dapoxetine treatment complained of lack of efficacy which was consistent with the main reasons for discontinuation of dapoxetine or SSRIs in phase 3 clinical trials [6, 25].

In the reported phase 3 trials, 64.9% of the study population were primarily categorized as patients with lifelong PE [4]. In our study, alongside primary PE incidence, we investigated other factors including, marital status, chronic prostatitis, intercourse frequency, prior use and reported efficacy of SSRIs, circumcision or penile dorsal nerve transection, ED and PE severity. While no correlation between efficacy of dapoxetine and the clinical factors listed above were identified, the severity of PE prior to treatment was found to be associated with the efficacy of dapoxetine.

Several factors may impact IELT and thus causing strong interpersonal differences which are reflected in the distribution of IELT values [26], including the performance of foreplay, the interval from last sexual experience, the gesture for sexual intercourse, the depth and force of thrusting, and the partner’s vaginal lubrication [27]. We compared patient clinical characteristics and found that most factors would not affect CGI-C, including PE category, effective SSRI use, and estimated IELT. McMahon et al. [28] reported PE category (*P* = 0.5) and estimated baseline IELT (*P* = 0.16) did not affect CGIC rating of “slightly better” in 285 Asia-Pacific men with PE. Patients with a lower PEDT score and a higher NITBE at baseline responded better to dapoxetine.

Interestingly, eIELT was not significantly different between” CGIC = 0″ and” CGIC≥1″ groups, suggesting that eIELT might be poorly estimated in the real-world setting. Lee et al. [7] compared stopwatch-determined IELT and eIELT in healthy men and found that eIELT was overestimated by approximately 1 min.

The stopwatch-determined IELT measured by female partner might not be suitable in real world practice, although it is considered the most objective measure for PE evaluation in clinical trials. Therefore, there is a need for a simple, relative and reliable tool for PE evaluation in real world practice. In this study, we have introduced NITBE as a measure for PE evaluation. We found that baseline NITBE could predict dapoxetine treatment efficacy, and therefore, might be a useful measure for PE evaluation and might be more accurate than eIELT.

Waldinger et al. [29] proposed NITBE in 1994 and found that NITBE between patient assessment and partner assessment was consistent. However, NITBE was not used in subsequent studies. As a very convenient method, it would be possible to evaluate the IELT in daily life and might be a useful tool for PE diagnosis and treatment evaluation in real word practice; however, further validation is required.

Our study has several strengths, including the analysis of co-existing clinical factors that potentially impact PE, and we firstly used NITBE to evaluate PE and found better role of NITBE on PE evaluation than eIELT. In further studies, we will compare NITBE with stopwatch-determined IELT in healthy men and patients with PE. Despite its novel findings, the present study does have some limitations. This was a retrospective, open-label study and the sample size was small relative to clinical trials; there might be some inevitable bias due to the retrospective nature. A further prospective study would be required to reduce bias and increase the strength. A further limitation of the study was that objective stopwatch-determined IELT is difficult to obtain accurately in real world practice, thus clinical trials are required to testify the impact of frequency of penile movement on NITBE. Therefore, patients were included in the study based on the PEDT score, and patient-reported measures included PEP, CGIC and NITBE. Besides although PEP questionnaire has been used in previous studies focusing on Chinese patients [30], the linguistic validation has not been performed.

## Conclusions

Dapoxetine treatment increased CGIC, eIELT and NITBE and was well-tolerated with an acceptable safety profile. Patients with less severe PE based on PEDT and higher NITBE seemed to have better efficacy with dapoxetine.

## Supplementary information

**Additional file 1.** Part of the database. Some information of the database, including IELT, NITBE and PEDT.

## Data Availability

Parts of the data are attached in supplementary file. The datasets used and/or analysed during the current study are available from the first author (Peng J & Fang D) and corresponding author (Zhang Z) on reasonable request.

## Abbreviations

CGIC: Clinical global impression of change
eIELT: Intravaginal ejaculation latency time
NITBE: Number of intravaginal thrusts before ejaculation
PE: Premature ejaculation
PEDT: Premature Ejaculation Diagnostic Tool
PEP: Premature ejaculation profile
